# Protein arginine methyltransferase 1 is a therapeutic vulnerability in multiple myeloma

**DOI:** 10.3389/fimmu.2023.1239614

**Published:** 2023-08-04

**Authors:** Hong Phuong Nguyen, Anh Quynh Le, Enze Liu, Annamaria Cesarano, Francesco DiMeo, Fabiana Perna, Reuben Kapur, Brian A. Walker, Ngoc Tung Tran

**Affiliations:** ^1^ Herman B Wells Center for Pediatric Research, Department of Pediatrics, Indiana School of Medicine, Indianapolis, IN, United States; ^2^ Melvin and Bren Simon Comprehensive Cancer Center, Division of Hematology and Oncology, School of Medicine, Indiana University, Indianapolis, IN, United States; ^3^ Center for Computational Biology and Bioinformatics, School of Medicine, Indiana University, Indianapolis, IN, United States; ^4^ Division of Hematology/Oncology, Department of Medicine, Indiana University School of Medicine, Indianapolis, IN, United States

**Keywords:** multiple myeloma, PRMT1, targeted therapy, relapsed/refractory myeloma, xenograft model

## Abstract

Multiple myeloma (MM) is a devastating plasma cell malignancy characterized by the expansion of aberrant monoclonal plasma cells in the bone marrow, leading to severe clinical manifestations and poor prognosis, particularly in relapsed/refractory cases. Identifying novel therapeutic targets is crucial to improve treatment outcomes in these patients. In this study, we investigated the role of the protein arginine methyltransferase 1 (PRMT1) in MM pathogenesis and explored its potential as a therapeutic target. We observed that PRMT1, responsible for most asymmetric di-methylation in cells, exhibited the highest expression among PRMT family members in MM cell lines and primary MM cells. Importantly, PRMT1 expression was significantly elevated in relapsed/refractory patients compared to newly diagnosed patients. High expression of PRMT1 expression was strongly associated with poor prognosis. We found that genetic or enzymatic inhibition of PRMT1 impaired MM cell growth, induced cell cycle arrest, and triggered cell death. Treatment with MS023, a potent PRMT type I inhibitor, demonstrated a robust inhibitory effect on the viability of primary cells isolated from newly diagnosed and proteasome inhibitor-relapsed/refractory patients in a dose-dependent manner. Suppression of PRMT1 downregulated genes related to cell division and upregulated genes associated with apoptosis pathway. We also found that genes related to immune response and lymphocyte activation were significantly upregulated in PRMT1-suppressed cells. Notably, the activation status of T cells was strikingly enhanced upon co-culturing with PRMT1-KO MM cells. *In vivo* studies using a xenograft model revealed that targeting PRMT1 by either CRISPR/Cas9-mediated knockout or MS023 treatment significantly attenuated MM tumor growth and prolonged the survival of tumor-bearing mice. Histological analysis further confirmed increased apoptotic cell death in MS023-treated tumors. Collectively, our findings establish PRMT1 as an indispensable and novel therapeutic vulnerability in MM. The elevated expression of PRMT1 in relapsed/refractory patients underscores its potential as a target for overcoming treatment resistance. Moreover, our results highlight the efficacy of MS023 as a promising therapeutic agent against MM, offering new avenues for therapeutic approaches in relapsed/refractory MM.

## Introduction

Multiple Myeloma (MM) is a severe hematological malignancy with typical CRAB (Calcium elevation, Renal insufficiency, Anemia, and Bone lesions) symptoms (1). Driven by intrinsic genomic abnormalities, MM progresses from a pre-malignant stage (monoclonal gammopathy of undetermined significance) to malignant disease (intramedullary and extramedullary MM) (2). Despite current advanced therapeutic treatment, relapsed patients are incurable (3). Thus, identifying novel targets vulnerable to MM is an unmet clinical need.

Protein Arginine Methyltransferases (PRMTs) catalyze the addition of one or two methyl groups onto the guanidine nitrogen atoms of arginine residues of proteins (4). PRMT1 is a type I PRMTs that is responsible for the majority of asymmetric di-methylated modifications in the cells (5). PRMT1 is a critical regulator of transcription, translation, RNA splicing, signal transduction, and protein homeostasis (6). Aberrant expression of *PRMT1* is strongly correlated with poor outcomes of both solid tumors and hematologic malignancies (7–9). However, how *PRMT1* contributes to MM pathology is virtually unknown.

Exploiting the publicly available transcriptomic data, we found that, among PRMTs, *PRMT1* expression is highest in MM cell lines and primary MM patient cells. Intriguingly, expression of *PRMT1* in relapsed/refractory patients is significantly higher than that of in newly diagnostic patient, indicating that *PRMT1* might be a vulnerability for relapsed/refractory MM. We also found that patients that highly express *PRMT1* are strongly associated with poor survival (both progression-free and overall survival). Suppression of *PRMT1* by genetic (CRISPR/Cas9-mediated knockout) or pharmaceutical (inhibitor) approaches impairs MM cell growth, arrests cell cycle, and enhances cell dead. Using xenograft models, we found that targeting *PRMT1* genetically or pharmaceutically attenuates the MM tumor growth and mitigates the tumor burdens *in vivo*. Additionally, a PRMT1 inhibitor strikingly decreased the viability of primary MM cells isolated from patients. Unbiased transcriptomic analysis showed that *PRMT1* suppression downregulated genes in the cell division pathway and upregulated the apoptosis signature. We also found a striking increases in genes related to the lymphocyte activation and immune response in *PRMT1* suppressed cells. Of note, we found *PRTM1*-suppressed cells induce T cell activation.

Collectively, we demonstrated that *PRMT1* is indispensable for MM cells, and targeting *PRMT1* provides a novel therapeutic approach to treating MM patients.

## Materials and methods

### Cell culture

MM cell lines JJN3, KMS11, and MM1S were cultured in RPMI-1640 medium (ThermoFisher Scientific, Waltham, MA, USA) supplemented with 10% fetal bovine serum (FBS, Corning), 2 mM L-glutamine, sodium pyruvate, and 50μM β-Mercaptoethanol (Sigma). Cells were maintained in an incubator with 5% CO2 at 37oC.

To generate Cas9-expressing MM cells, we infected MM cells with lentivirus carrying Cas9 and mCherry as a reporter (Addgene 70182). mCherry-positive cells were sorted out by FACS sorter (FACSFusion, BD). To generate inducible knockout MM cells, Cas9-expressing cells were infected with lentivirus carrying sgRNAs and puromycin-resistant gene (Addgene, 104321). Infected cells were selected with a medium containing puromycin (0.5-1μg/ml) for at least one week. For inducing the expression of sgRNAs, cells were cultured in the medium supplemented with doxycycline (1μg/ml) into the culture medium. 3-4 days after inductions, cells were harvested for other cellular assays.

Human CD138+ cells from MM patients (newly diagnosed and relapsed/refractory patients that were treated with proteasome inhibitors) were generously provided by the Indiana Myeloma Registry. Cells were cultured in MM medium and treated with MS023 (MedChemExpress) at different concentrations. Cell viability was assessed after 48h of treatment.

CB-CD34 cells were cultured in SFEMII media supplemented with cytokines (stem cell factor (SCF), FLT-3 ligand, interleukin (IL)-3, IL-6, IL-9, and IL-11) (Peprotech) for maintaining and experiments.

### Western blot

Cells were harvested, washed with PBS once, and resuspended into RIPA buffer (Sigma) (10^6^ cells/ml) supplemented with proteasome inhibitor cocktail (Millipore). Cells were incubated on ice for 30 minutes and spun at 20,000 g for 30 minutes at 4°C. The supernatant was collected as whole cell lysate. Protein was quantified by standard Bradford assay (Biorad). 10-40μg of proteins was used for western blot following a standard protocol. PRMT1 protein was detected by rabbit anti-human PRMT1 antibody (07-404, Millipore). Mono-methyl arginine antibody (8015S) and asymmetric di-methyl arginine antibodies (13522S) were purchased from cell signaling. GAPDH (Proteintech) was used as a loading control.

### Coculture of T cells and myeloma cells

T cells were isolated from healthy donor using CD3 magnetic beads (Miltenyi Biotec). CD3 T cells (10^6^ cells) were activated with 30μl of CD3/CD28 beads (Invitrogen) in T cell medium (X-VIVO medium +5% FBS+1% penicillin/streptomycin supplemented with human IL2 400U/ml (Peprotech) for 3-4 days. Magnetic beads were removed, and T cells were stained with cell trace (CFSE, Invitrogen), following manufacturer’s manual. Irradiated myeloma cells were plated into the T cell culture at a ratio of 1 myeloma: 2 T cells. For non-contact co-culture of T cells and MM cells, MM cells were cultured in the upper chamber of transwell insert (pore size, 3 μM, #09-761-80, Thermo Fisher) in U-bottom 96 well plate. 4 days activated T cells were cultured in the lower chambers of 96-well transwell plate. 48 hours later, cells were stained with CD3-FITC, CD25-APC, and CD69-PE (Biolegend), and analyzed by flow cytometry (Atune NxT, Invitrogen).

### Cell cycle analysis

Cells were washed twice with PBS and fixed with 70% ethanol overnight at 4°C. Fixed cells were washed 3 times with cold PBS and resuspended into a staining buffer containing 0.5 μg/ml RNase A (Millipore) and Propidium Iodide (PI, 50 μg/ml, Sigma). Cells were incubated at 4°C overnight and analyzed by flow cytometry (Atune NxT, Invitrogen).

### Cell apoptosis

Cells were harvested and washed twice with cold PBS buffer prior to resuspending in binding buffer (0.1M Hepes pH 7.4, 1.4M NaCl, 25 mM CaCl2 solution). The cells were stained with Annexin V-FITC (Biolegend) and PI for 15min at RT. The cells were analyzed by using flow cytometry (Atune NxT).

### Cellular inhibition assay

Cells were seeded in 96-well culture plates at 2x10^4^ cells/well for overnight. The cells were treated with drugs at different concentrations for 48 h. Cell viability was measured using a CellTiter-Glo kit 2.0 (Promega). The inhibition rates of the test compounds were calculated based on the dimethyl sulfoxide (vehicle)-treated group and negative control (Media only). The inhibitory activity of the tested compounds was evaluated by a dose–response curve (DRC) analysis in triplicates. The IC50 values were calculated using Prism v9.5.0 (GraphPad Software Inc., La Jolla, CA).

### RNA sequencing

Wildtype JJN3, PRMT1-KO JJN3, and MS023 treated JJN3 cells were harvested for RNA isolation. Total RNA was sequenced to >60 million read pairs were sequenced per sample. Fastqc was used for analyzing read quality while trimmomatic was utilized for removing identified low-quality reads and adapters. Passing reads were further aligned to Hg38 genome and Gencode V35 (hg38) genomic annotation by STAR3. ‘Two-pass’ mode was applied to ensure maximum alignment quality. On average 82.87% of reads were passed and uniquely aligned. Transcriptome was quantified by Salmon4 (Quasi-mapping mode) using Hg38 reference Gentrome (a combined reference of Hg38 reference genome and Gencode V35 transcriptome) with the following parameters (K-mer=31, standard EM algorithm and ‘ValidateMappings’). Quantified read counts were further normalized to transcript-per-million (TPM) on transcript level and gene-level.

### Differential gene expression

Log2(TPM+1) transformation was conducted to rescale expression levels. Limma was subsequently applied to identify differentially expressed genes and estimate their log-fold changes. Limma5 conducted moderated t-tests to normalize the batch effect and correct the differential expression. A set of thresholds with a false discovery rate (FDR)<0.05, absolute log2-fold-change >0.48 (fold change >1.4 or <0.71), and log2(TPM+1)>1 in comparisons was applied to identify significantly and differentially expressed genes.

### Pathway analysis

Pathway enrichment analysis was performed by GSEA (10) for differentially expressed genes. ‘Hallmark cancer’, ‘KEGG’ and ‘Gene Ontology Biological Process’ were used as the pathway sets from MGigDB (11). Significantly dysregulated pathways were further identified with P<0.05. Bubble plots were generated using “Clusterprofiler” (12).

### Xenograft model

NOD. Cg-Prkdcscid Il2rgtm1Wjl/SzJ (NSG) mice were bred and maintained in the *In Vivo* Therapeutics Core (IVT) at Indiana University School of Medicine. All the experiments were performed according to the guidelines of the Institutional Animal Care and Use Committee.

Mycoplasma-negative MM cell lines (5x10^6^ cells) were resuspended in 200ul phosphate buffer saline (PBS) and injected subcutaneously into the right flank of NSG mice (8-10 weeks old). Tumor growth was monitored using calipers. Tumor-bearding mice were sacrificed when tumor volume reached 2000mm^3^.

For MS023 treatment, 10 days after tumor inoculation, mice were randomized based on their tumor volumes. A total of four doses of MS023 80mg/kg (MedChemExpress) was intraperitoneally injected into the mice every other day. Tumor growth was monitored using calipers. Tumor-bearding mice were sacrificed when the tumor volume reached 2000mm^3^. When the mice in the vehicle group reached the humane endpoint, tumors were harvested, paraffin-embedded, sectioned, and stained for Hematoxylin & Eosin, and cleaved Caspase 3 (Cell Signaling, # 9664S). The percentage of positive area for the cleaved Caspase 3 was quantified by using Image J.

### Statistical analysis

Statistical tests were performed using Prism 9.0 (GraphPad) using a paired two-tailed Student’s t-test, or one-way ANOVA, or two-way ANOVA. ****P <0.0001; *** P<0.001, ** P<0.01. For each experiment, at least three independent experiments were performed. Data are shown as mean values. The number of biological replicates for each experiment is shown in the figure legends and shown as data points in the figures.

### Survival analysis

Survival analysis was conducted using annotations of 598 newly diagnosed MM samples in the MMRF compass dataset (IA16). The Kaplan-Meier plot was used for displaying the survival probability while the log-rank test was performed for measuring the survival difference.

## Results

### *PRMT1* is highly expressed in MM cells and is associated with poor prognosis

**Figure 1:**
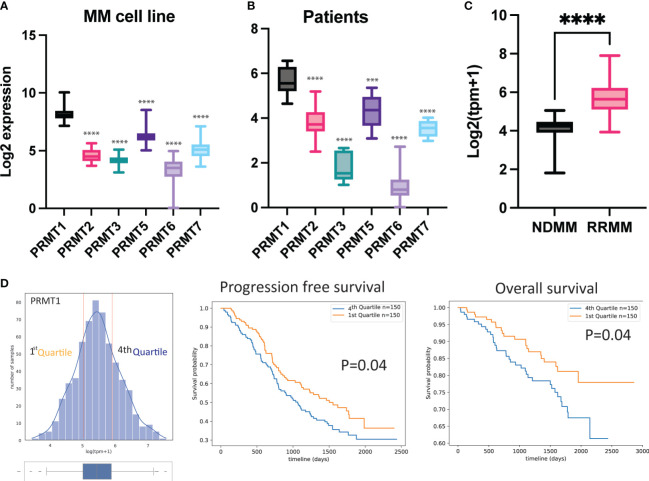
*PRMT1* is strongly associated with poor prognosis of MM patients. Expression of PRMTs in MM cell line **(A)** and primary MM patient cells **(B)**. **(C)** Expression of *PRMT1* in newly diagnosed patient (NDMM) and relapsed/refractory patients (RRMM). TPM+1: transcripts per million. Data are shown as mean value. P values were calculated using one-way Anova test (compared to *PRMT1* group) are shown in the graph or as ****P<0.0001 and ***P<0.001. **(D)** MM patients were divided into 2 groups based on expression of *PRMT1*: low expression (1^st^ Quartile) and high expression (4^th^ Quartile). Progression-free survival (middle) and overall survival (right) of patients with different expression level of *PRMT1*. P-values were derived from log-rank test.

Aberrant expression of protein arginine methyltransferases (PRMTs) has been strongly associated with many cancers. We leveraged publicly available transcriptomic data from the Cancer Cell Line Encyclopedia and analyzed the expression levels of PRMTs in MM cell lines. Our analysis revealed that *PRMT1* is the most highly expressed gene among the investigated PRMTs across a wide range of MM cell lines (**Figure 1A**). Furthermore, we examined transcriptomic data from MM patients obtained from the Multiple Myeloma Research Foundation CoMMpass study and found that *PRMT1* is expressed at significantly higher levels than the other members (**Figure 1B**). Interestingly, the expression level of *PRMT1* was significantly higher in relapsed/refractory patients compared to newly diagnosed patients (**Figure 1C**), suggesting potential functions of *PRMT1* in relapsed/refractory development. Importantly, patients with high expression levels of PRMT1 were associated with poor survival rates, as indicated by both progression-free and overall survival analyses (**Figure 1D**). These findings strongly suggest that *PRMT1* may play an important role in the progression of MM.

### CRISPR/Cas9-mediated knockout of PRMT1 impairs MM cell growth, arrests cell cycle, and enhances cell death

**Figure 2:**
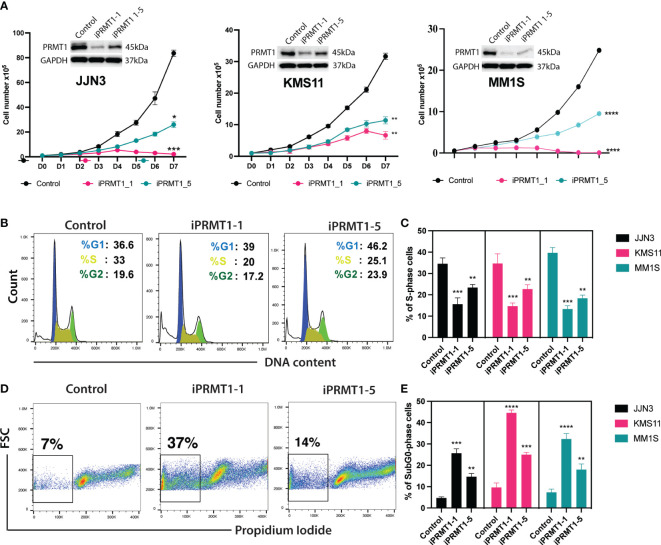
Knockout of PRMT1s impairs MM cell growth, arrests cell cycle, and induces cell death. **(A)** Growth curves of 3 different MM cell lines (JJN3, KMS11, and MM1S) upon knockout of *PRMT1* by two different sgRNAs (iPRMT1_1, and iPRMT1_5). Western blot data (on top of each growth curve) shows the knockout efficiency of *PRMT1* in MM cells for each sgRNAs. **(B)** Representative FACS blot shows cell cycle profile of MM cell lines upon knockout of *PRMT1* by two different sgRNAs. **(C)** Bar graph depicts mean values of percentage of S phase cells from three independent biological replicates. **(D)** FACS blot describes the representative cell death (Sub G0 phase) profile of MM cell lines. **(E)** Bar graph shows the mean values of percentage of Sub-G0 phase cells from biological triplicates. P values were calculated using one-way Anova **(C, D)** or two-way Anova **(A)** tests are shown as ****P<0.0001, ***P<0.001, **P<0.01 and *P<0.05. Data represent as means of three independent replicates.

To evaluate the functions of *PRMT1* in multiple myeloma (MM) cells, we generated inducible knockout MM cell lines using the CRISPR/Cas9 system. Initially, we established MM cell lines stably expressing the Cas9 gene. Subsequently, we designed a set of single guide RNAs (sgRNAs) targeting *PRMT1* using the CrispRGold program (13) and validated their targeting efficiencies using T7E1 assay and western blot (**Supplementary Figure 1**). We selected the two most efficient sgRNAs (sgPRMT1-1 and sgPRMT1-5) to generate inducible knockout cell lines. The sgRNAs were cloned into an inducible lentiviral vector. Cas9-expressing MM cells were infected with lentivirus expressing inducible sgRNAs. Stable cell lines were selected prior to experiments. To knockout *PRMT1*, we supplemented doxycycline (Dox) into the medium. Three days later, cells were used for functional assays. In the presence of Dox, sgPRMT1-1 induced the deletion of PRMT1 protein more efficiently than sgPRMT1-5 in all tested cell lines (**Figure 2A**). Notably, we observed that the knockout of *PRMT1* by sgPRMT1-1 exhibited a stronger inhibitory effect on MM cell growth than sgPRMT1-5, indicating a dose-dependent effect of PRMT1 (**Figure 2A**). We further assessed the cell cycle profile of *PRMT1*-KO MM cells and found that the deletion of *PRMT1* significantly reduced numbers of cells in S phase (**Figures 2B, C**). Additionally, the proportion of sub-G_0_ phase cells in *PRMT1*-KO cells was strikingly elevated compared to control cells (**Figures 2D, E**). We also performed Annexin V staining and found that deletion of PRMT1 robustly induced both cell death and apoptotic cell (**Supplemental Figure 2**). Collectively, these data demonstrated that deletion of *PRMT1* in MM cells resulted in impaired cell growth, arrested cell cycle, and enhanced cell death.

### MS023, a type I PRMTs inhibitor, recapitulates the *PRMT1* KO cells *in vitro*

**Figure 3:**
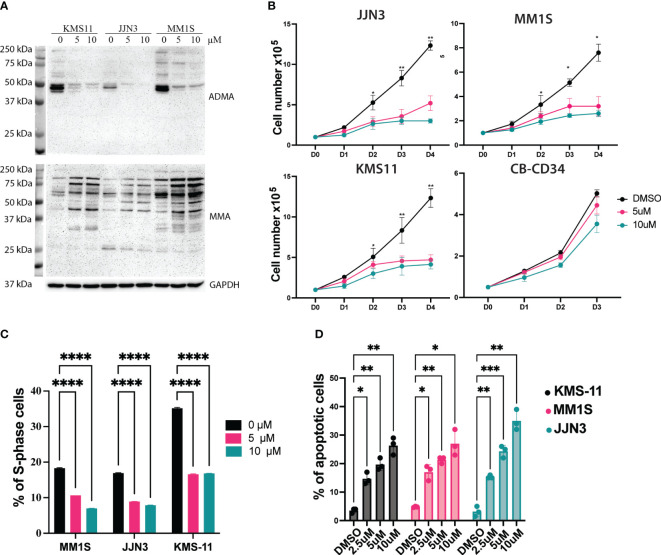
Suppression of the enzymatic activity of *PRMT1* exhibits a strong anti-MM effect. **(A)** Western blot showing the effects of MS023 on the total asymmetric arginine di-methylation (ADMA) and Arginine mono-methylation (MMA) in three different MM cell lines with different concentrations for 3 days. **(B)** growth curve of MM cell lines and human CD34+ cells upon treated with MS023 with different concentrations. **(C)** Summary of S-phase percentage of MM cell lines upon treated with MS023 for 3 days. **(D)** Bar graph summarizes the apoptotic profiles of MM cell lines upon treated with MS023. Data are shown as mean values of biological triplicates. P values were calculated using one-way Anova **(C, D)** and two-way Anova **(B)** tests are shown in the graph or as ****P<0.0001, ***P<0.001, **P<0.01 and *P<0.05.

CRISPR/Cas9-mediated targeting of *PRMT1* might not be clinically relevant so we sought to exploit a PRMT1 inhibitor that could be used to target *PRMT1 in vivo*. MS023 is a specific inhibitor for type I PRMTs and has a strong affinity to PRMT1 (14). After 3 days of treatment, MS023 exhibits a strong inhibitory effect for global arginine-asymmetric di-methylation as well as accumulation of arginine mono-methylation in MM cells (**Figure 3A**). Next, we assessed the effects of MS023 in MM cells. MM cell lines were treated with MS023 at different concentrations. Cells were harvested at different time points to analyze cell growth, cell cycle and cell death. We found that MS023 significantly impaired the MM cell growth in the dose-dependent manners. Notably, MS023 only exhibits a mild inhibitory effect on the human CD34^+^ cells (**Figure 3B**). Consistent with the *PRMT1* KO data, we found that suppression of the enzymatic activity of PRMT1 significantly decreased proportions of S-phase cells (**Figure 3C**, **Supplemental Figure 3B**). When staining with Annexin V, we found that MS023 induced apoptotic cells in dose-dependent manner (**Figure 3D**, **Supplemental Figure 3**). Collectively, these data indicate that *PRMT1* plays an indispensable role in the growth and survival of MM cells.

### Suppression of *PRMT1* induces cell death in primary cells from MM patients

**Figure 4:**
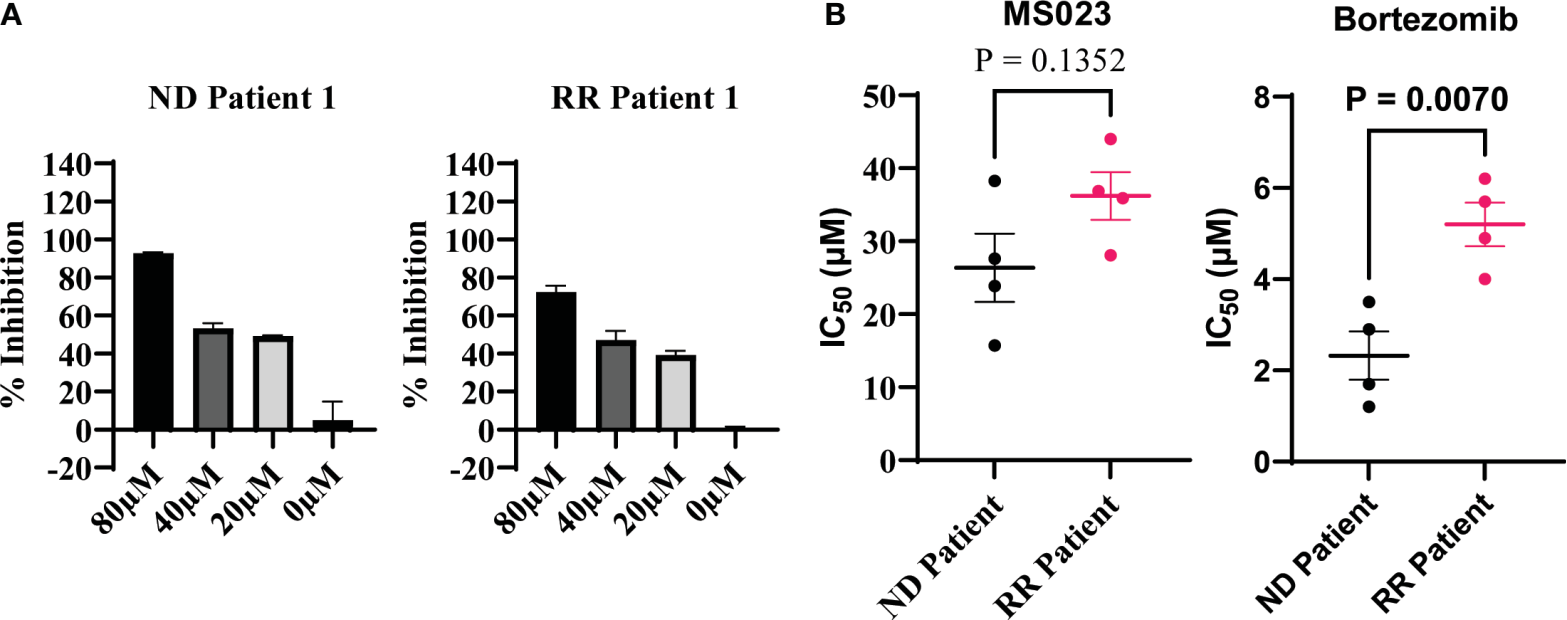
Inhibitory effects of MS023 on primary cells isolated from MM patients. **(A)** Cell viability assay for primary cells treated with MS023 at different concentration for 2 days: newly diagnosed patient (ND) and relapsed/refractory patients (RR). **(B)** IC50 (μM) of MS023 indicates the sensitivity of primary patients with MS023. Each dot represents for one patient (other primary patient samples in **Supplementary Figure S4B**).

Next, we investigated the inhibitory potential of MS023 in primary cells obtained from patients diagnosed with multiple myeloma (MM). Considering the limited ex vivo proliferation of primary MM cells in standard culture medium, we employed the CellTiter-Glo luminescent kit to evaluate cell viability based on ATP production. We procured primary MM cells from four newly diagnosed patients and four patients who had relapsed after prior treatment with a proteasome inhibitor. These cells were treated with varying concentrations of MS023, and subsequent cell viability assays were performed to assess the impact of the compound on MM cell survival. We found that MS023 exerted a robust dose-dependent inhibitory effect on both newly diagnosed and relapsed/refractory primary MM cells (**Figure 4A**). Notably, the patient-derived cells displayed diverse sensitivity to MS023, as depicted in **Supplementary Figure 4**. Furthermore, we determined the IC50 values of MS023 and bortezomib for the patient cells. As expected, cells isolated from relapsed patients exhibited considerable resistance to bortezomib. While there was a modest trend towards increased resistance to MS023 in relapsed cells compared to newly diagnosed cells, this difference was not statistically significant (**Figure 4B**)

Collectively, these findings underscore the potent inhibitory activity of MS023 on primary MM cells, highlighting its potential as a promising therapeutic agent in the treatment of MM, particularly in patients with relapsed/refractory disease who have exhibited resistance to conventional therapies.

### Global transcriptomic change in *PRMT1* suppressed cells

**Figure 5:**
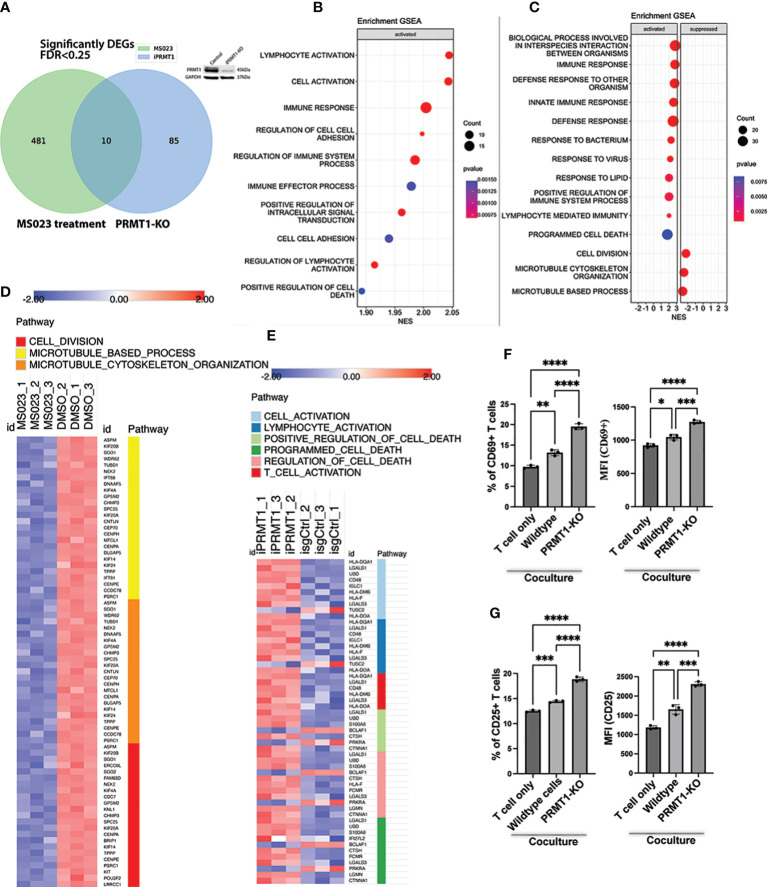
Transcriptional impacts of *PRMT1* suppression in MM cell lines. **(A)** Venn diagram shows the numbers of differentially expressed genes (DEGs) in MM cells upon *PRMT1*-KO (using iPRMT1_1) or treated with MS023. Western blot on the top of the Venn diagram proves the knockout efficiency of PRMT1. **(B)** Gene set enrichment analysis (GSEA) of DEGs in PRMT1-KO cells. **(C)** GSEA of DEGs in MM cells treated with MS023. Heat map presents the expression of DEGs in MS023-treated MM cells **(D)** and *PRMT1*-KO cells **(E)**. **(F)** Graph depicts the percentage of CD69+ T cells (left) and the mean fluorescence intensity (MFI) of CD69 (right) either cultured alone or cocultured with MM cells (wildtype and *PRMT1*-KO cells). **(G)** Bar graph describes the percentage of CD25+ T cells (left) and the MFI of CD25 (right) either cultured alone or cocultured with irradiated MM cells (wildtype and *PRMT1*-KO cells) for 2 days. Data are shown as mean values. P values calculated using one-way Anova test and are shown in the graph or as ****P<0.0001, ***P<0.001, **P<0.01 and *P<0.05.

To understand how *PRMT1* regulates MM growth, we assessed the global transcriptomic change of the JJN3 cells upon either deletion of *PRMT1* or treatment with MS023. To delete *PRMT1*, cells were induced with doxycycline for 3 days, and the harvested for RNA sequencing. After 3 days of induction, *PRMT1* was efficiently deleted (**Figure 5A**). We also treated cells with MS023 for 3 days and collected treated cells for RNA sequencing. 3 days of MS023 treatment showed striking reduction of arginine di-methylation and accumulation of arginine mono-methylation in MM cells (**Figure 3A**). From RNA sequencing, we found that while there were 491 differentially expressed genes (DEGs) in the cells treated with MS023 compared to DMSO-treated cells, there were only 95 DEGs in *PRMT1*-KO cells compared to *PRMT1* wildtype cells, with 10 genes overlapping (**Figure 5A**). Pathway analysis and gene set enrichment analysis showed that MS023 significantly suppressed genes associated with cell division: G1/S transition (CDC7), spindle assembly (ERCC6L and ASPM), centromere structure and function (CENPA, CENPE, and LRRCC1), Kinetochore complex (SPC25 and KNL1), mitotic regulators (TPPP, PSRC1, FAM83D, SGO1, and SGO2), Kinesin family (KIF4A, KIF20A, KIF20B, and KIF14 proliferation-related genes (KIT and POU3F2) (**Figure 5C**, **Supplementary Figure 5D**). Both *PRMT1*-KO and MS023-treated cells activated genes related to the cell death programs (**Figures 5B, C**). Intriguingly, we found pathways related to immune responses, lymphocyte activation, and T cell activation were significantly upregulated in *PRMT1*-suppressed MM cells (**Figures 5B–E**). We also found that MHC class II genes (HLA-DQ, DM, and DO) were significantly upregulated upon PRMT1 depletion. This data indicates that PRMT1 might play a role in suppressing the antigen presentation of MM cells to immune cells. Collectively, these data led us to the hypothesis that *PRMT1* might play a role in T cell suppression upon interacting with MM cells. To test this hypothesis, we performed the T cell and MM cell coculture experiments (15). Briefly, human T cells were activated and cultured in the medium containing human IL2 for 4 days. We stained activated T cells with Cell-trace dye (CSFE) and then co-cultured with either irradiated wildtype JJN3 or *PRMT1*-KO JJN3 cells with a ratio of 1 MM cells and 2 T cells. After 2 days of co-culture, we assessed the proliferation and activation status of T cells. CD69 (16, 17) and CD25 (18, 19) are well-known marker of activated T cells. Thus, we will use these surface markers to assess the activation status of T cells upon co-culture with MM cells. We found that coculture with MM cells (either wildtype or *PRMT1*-KO) did not enhance the proliferation of activated T cells compared to T cell only (**Supplementary Figure 5A**). However, when we looked at the expression of CD69 and CD25, T cell activation markers, we found that the expression of CD25 and CD69 (percentage of positive cells and the mean fluorescence intensity, MFI) in T cells cocultured with MM cells were strongly enhanced in comparison to that of T cell cultured without MM cells. Notably, *PRMT1*-KO cells induced the expression of both CD69 and CD25 significantly stronger than the wildtype cells (**Figures 5F, G**, **Supplementary Figure 5B**). To address whether this effect is cell contact-dependent, we set up a co-culture experiments using the trans-well system. Briefly, the activated T cells were cultured in bottom chamber of transwell plate, while MM cells (wildtype or PRMT1-KO) were seeded on the upper chamber. With this setup, there was no direct interaction of MM cells and T cells. After 2 days of co-culture, we assessed the activation markers of T cells (CD25 and CD69). We found that there was no significant difference in the activation status of T cell co-cultured with MM cells compared to T cells cultured without MM cells (**Supplementary Figure 6**). Collectively, these data suggest the potential role of *PRMT1* inhibition in T cell-mediated immunotherapy.

### PRMT1 inhibition exhibits anti-MM activity *in vivo*

**Figure 6:**
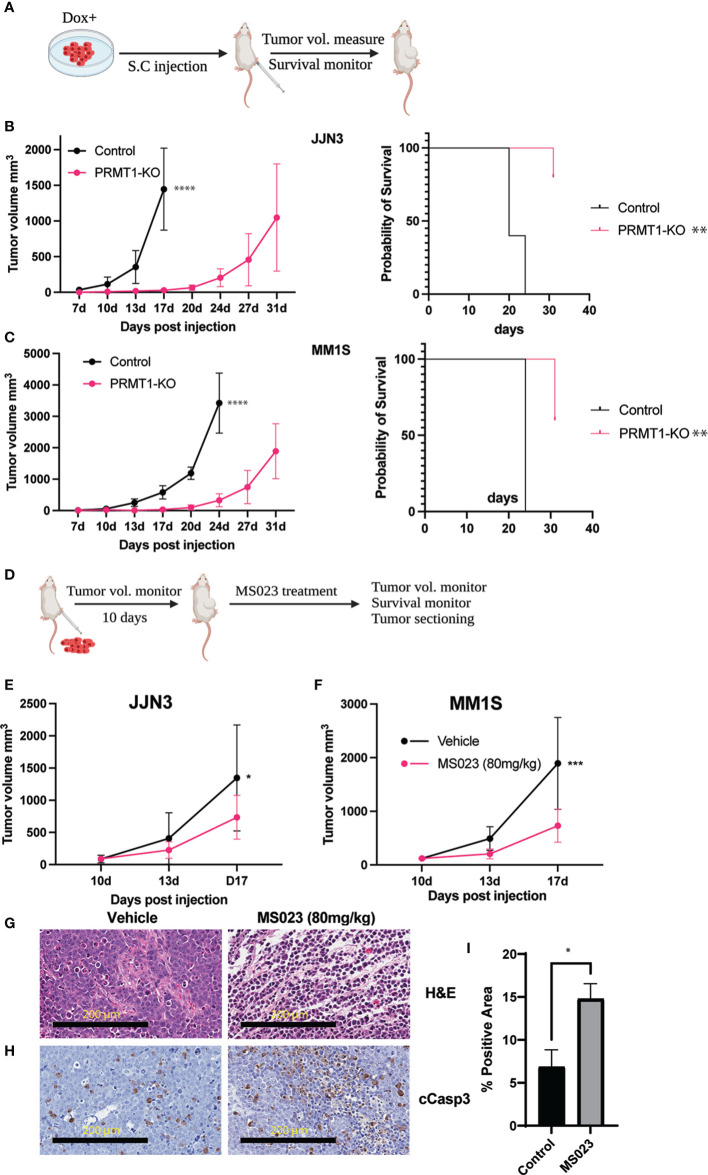
Suppression of *PRMT1* attenuates MM tumor growth *in vivo*. **(A)** Experimental scheme of MM xenograft model for *PRMT1*-KO cells. Tumor volumes of in the mice injected with JJN3 **(B)** or MM1S **(C)** WT cells (control) and *PRMT1*-KO cells (left, n=5), Kaplan-Meier survival curve of mice injected with WT and *PRMT1*-KO cells. **(D)** Experimental scheme of MM xenograft model for MS023 treatment. Tumor volumes at different time points of vehicle group (N=5) and MS023-80mg/kg group (n=5) in mice injected with JJN3 cells **(E)** and MM1S **(F)** cells. **(G)** Hematoxylin and Eosin (H&E) staining of tumor sections. **(H)** immunohistochemistry staining for the cleavage of Caspase 3 (cCasp3). **(I)** Bar graph summarizes the cCasp3 positive area in the stained slides. Data are shown as mean values. P values calculated using two-way Anova test and T test **(I)** are shown in the graph or as ****P<0.0001, ***P<0.001, **P<0.01 and *P<0.05.

We have successfully demonstrated the essential role of *PRMT1* in MM cells *in vitro*. Building upon these findings, we aimed to evaluate the *in vivo* anti-MM effects of *PRMT1* suppression. To accomplish this, we employed a well-established xenograft model by implanting MM cell lines into the flank of NOD-scid IL2Rγ^null^ (NSG) mice. Cells were expanded, induced with Dox, and implanted into the flank of NSG mice (**Figure 6A**). Tumor volumes were closely monitored until the humane endpoints were met. Our experiments utilizing different MM cell lines consistently showed that knockout of PRMT1 significantly delayed MM tumor progression *in vivo* and extended the survival of tumor-bearing mice (**Figures 6B, C**, **Supplementary Figure 6**).

Subsequently, we assessed the therapeutic efficacy of MS023 using the same xenograft model. After expanding MM cells and implanting them into the flank of NSG mice, we initiated drug treatment based on tumor size 10 days post-inoculation (**Figure 6D**). Notably, treatment with MS023 at a dose of 80 mg/kg resulted in significant suppression of tumor growth *in vivo* (**Figures 6E, F**). To gain further insights into the underlying mechanisms, we collected tumors from both the vehicle and MS023 treatment groups for histological examination. Hematoxylin and eosin staining revealed disrupted extracellular matrix and cytoplasmic structures in MS023-treated tumors (**Figure 6G**). Moreover, we observed an enriched signal of cleaved Caspase 3 in the MS023-treated tumors (**Figures 6H, I**), indicating induction of MM cell apoptosis and subsequent suppression of tumor growth by MS023 *in vivo*.

In conclusion, our study highlights *PRMT1* as a novel vulnerability in MM cells. Notably, *PRMT1* is highly expressed in relapsed/refractory MM patients, further emphasizing its clinical relevance. Targeting *PRMT1* presents a promising therapeutic strategy for the treatment of relapsed/refractory MM, offering new avenues for effective interventions in this challenging clinical context. Further investigations are warranted to elucidate the precise mechanisms underlying *PRMT1*-mediated MM pathogenesis and to optimize the therapeutic potential of *PRMT1* inhibition in MM management.

## Discussion

Our study provides compelling evidence for the crucial role of *PRMT1* in the pathogenesis of MM and highlights its potential as a therapeutic target. We are the first to show the direct link of *PRMT1* in multiple myeloma. We found that *PRMT1* is the highest expressed gene among PRMTs in MM cell lines as well as primary patient cells. Importantly, elevated *PRMT1* expression is strongly correlated with poor prognosis in MM patients, further emphasizing the clinical significance of gene. Notably, we observed a substantial increase in *PRMT1* expression in relapsed/refractory patients compared to newly diagnosed patients. This finding suggests that *PRMT1* may contribute to disease progression and treatment resistance, making it an attractive target for therapeutic intervention.

Emerging evidence has shown the direct link of PRMTs in cancer biology (7, 20). *PRMT1* is the most predominant enzyme in the family and plays a multifaced roles in both normal hematopoiesis and hematological malignancies (20). We previously showed that *PRMT1* regulates the splicing via directly methylating RBM15 in acute megakaryoplastic leukemia (21). We also demonstrated that *PRMT1* regulates the megakaryopoiesis via methylated DUSP4 leading to DUSP4 degradation and activation of p38 MAPK (22). *PRMT1* interacts with BTG2 to modulate the pre-B cell differentiation via methylation of CDK4 (23). Although *PRMT1* is crucial for the mature B-cell activation and differentiation, but not for their development and distribution in the mouse spleen. Moreover, *PRMT1* is essential in B cells for several processes involved in humoral immunity (24) and for their functionality, as *Prmt1^−/−^* mice display defects in the antibody response from T-cell-independent, but not from T-cell-dependent, antigen stimulation (25). Along this line, we found that *PRMT1* plays an important role in regulating the activation of T cells upon co-culturing with MM cells.

Our study for the first time showed the important role of PRMT type I in MM progression. Among the PRMT family members, *PRMT5*, a type II PRMT, has been identified as a known vulnerability in MM cells. However, the roles of other PRMTs in MM pathology remain largely unknown. *PRMT5* targets TRIM21 leading to inhibition of NF-kB pathway, a pathological pathway of MM cells (26). It has been reported that PRMT5 regulates pyroptosis via inhibiting the CASP1 in myeloma cells (27). Further study will focus on identifying the underlying mechanisms on how *PRMT1* controls the pathogenesis of MM.

To investigate the functional role of *PRMT1* in MM, we employed genetic knockout and enzymatic inhibition approaches. Our results revealed that loss of *PRMT1* function significantly impairs cell growth, induces cell cycle arrest, and enhances cell death. These findings highlight the critical involvement of *PRMT1* in promoting MM cell proliferation and survival. Moreover, our study provides mechanistic insights into the underlying processes regulated by *PRMT1*, suggesting its involvement in key cellular pathways associated with MM pathogenesis. Our unbiased transcriptomic analysis demonstrated that PRMT1 regulates key genes related to cell division in MM cells. PRMT1 is known to directly methylated cell cycle regulators in different cell contexts. PRMT1 methylates CDK4, preventing formation of CDK4-cyclin-D3 complex and cell cycle progression in pre-B cells (23). Methylation of E2F1 by PRMT1 hinders the PRMT5-mediated methylation, augmenting E2F1-dependent apoptosis in SAOS2 cells (28). Our data, in contrast, demonstrated that PRMT1 is critical for cell cycle of MM cells, indicating the cell-dependent functions of PRMT1. We will identify direct substrates of PRMT1 in MM cells in our future works.

Interesting, we found several pathways related to immune response, lymphocyte activation, and T cell activation were significantly upregulated upon PRMT1 suppression. The fact that the expression of the MHC class II genes was increased in PRMT1-KO cells indicates that PRMT1 might play a role in suppression of antigen presentation to T cells and other immune cells. Further studies to elucidate functions of PRMT1 in antigen presentation or the efficacy of targeting PRMT1 in immunotherapy is greatly interested.

Importantly, we evaluated the therapeutic potential of targeting *PRMT1* in MM using *in vivo* xenograft models. Through pharmacological inhibition of *PRMT1* with MS023, we observed a significant suppression of MM tumor growth *in vivo*. Histological analysis of the treated tumors revealed disrupted extracellular matrix and cytoplasmic structures, accompanied by increased apoptotic cell death. These findings underscore the effectiveness of *PRMT1* inhibition as a promising strategy for MM therapy, particularly in relapsed/refractory cases where alternative treatment options are limited. GSK3368715 is a potent oral PRMT type I inhibitor that exhibits strong anti-cancer activity *in vivo* in many preclinical models (29–31). This inhibitor was used in the clinical trial for solid tumors and diffused large B-cell lymphoma (NCT03666988). Our future study will exploit this inhibitor in xenograft model and patient-derived xenograft model.

The identification of *PRMT1* as a vulnerability in MM opens new avenues for therapeutic interventions. Targeting *PRMT1* not only impacts cell growth and survival but also holds the potential to overcome treatment resistance observed in relapsed/refractory patients. Future studies should focus on elucidating the precise molecular mechanisms by which *PRMT1* contributes to MM pathogenesis, exploring its crosstalk with other signaling pathways, and assessing the efficacy of PRMT1 inhibitors in combination with existing therapeutic modalities.

## Data availability statement

The datasets presented in this study can be found in online repositories. The names of the repository/repositories and accession number(s) can be found below: https://www.ncbi.nlm.nih.gov/genbank/, GSE234807.

## Ethics statement

The animal study was reviewed and approved by Institutional Animal Care and Use Committee.

## Author contributions

NTT conceived and developed the project. NTT, HN, and AL designed, performed, and analyzed experiments. EL, and BW oversaw and analyzed RNA sequencing data. AC, FD, and FP provided cell lines and assisted with T cell co-culture. RK assisted the revision. NTT wrote the manuscript. All authors contributed to the article and approved the submitted version.
