# Corrigendum: Protein arginine methyltransferase 1 is a therapeutic vulnerability in multiple myeloma

**DOI:** 10.3389/fimmu.2023.1334733

**Published:** 2023-11-15

**Authors:** Hong Phuong Nguyen, Anh Quynh Le, Enze Liu, Annamaria Cesarano, Francesco DiMeo, Fabiana Perna, Reuben Kapur, Brian A. Walker, Ngoc Tung Tran

**Affiliations:** ^1^Herman B Wells Center for Pediatric Research, Department of Pediatrics, Indiana School of Medicine, Indianapolis, IN, United States; ^2^Melvin and Bren Simon Comprehensive Cancer Center, Division of Hematology and Oncology, School of Medicine, Indiana University, Indianapolis, IN, United States; ^3^Center for Computational Biology and Bioinformatics, School of Medicine, Indiana University, Indianapolis, IN, United States; ^4^Division of Hematology/Oncology, Department of Medicine, Indiana University School of Medicine, Indianapolis, IN, United States

**Keywords:** multiple myeloma, PRMT1, targeted therapy, relapsed/refractory myeloma, xenograft model

In the published article, there was an error in affiliation 1. Instead of “Well Center for Pediatric Research, Department of Pediatrics, Indiana University School of Medicine, Indianapolis, IN, United States”, it should be “Herman B Wells Center for Pediatric Research, Department of Pediatrics, Indiana School of Medicine, Indianapolis, United States.”.

The authors apologize for this error and state that this does not change the scientific conclusions of the article in any way. The original article has been updated.

