# Supplementary material for: Protein arginine methyltransferase 1 is a therapeutic vulnerability in multiple myeloma
Source: Front Immunol. 2023 Aug 4;14:1239614. doi: 10.3389/fimmu.2023.1239614 (PMC10436492; doi:10.3389/fimmu.2023.1239614)
Supplement: Supplementary file 1 [file DataSheet_1.docx]

Supplementary Material

Protein Arginine Methyltransferase 1 is therapeutic vulnerability in Multiple Myeloma

**Hong Phuong Nguyen^1^, Anh Quynh Le^1^, Enze Liu^2,3^, Annamaria Cesarano^4,5^, Francesco DiMeo^4,5^, Fabiana Perna^4,5^, Brian A. Walkers^2,3^, and Ngoc Tung Tran^1*^**

*** Correspondence:** Ngoc Tung Tran: tungtran@iu.edu

**
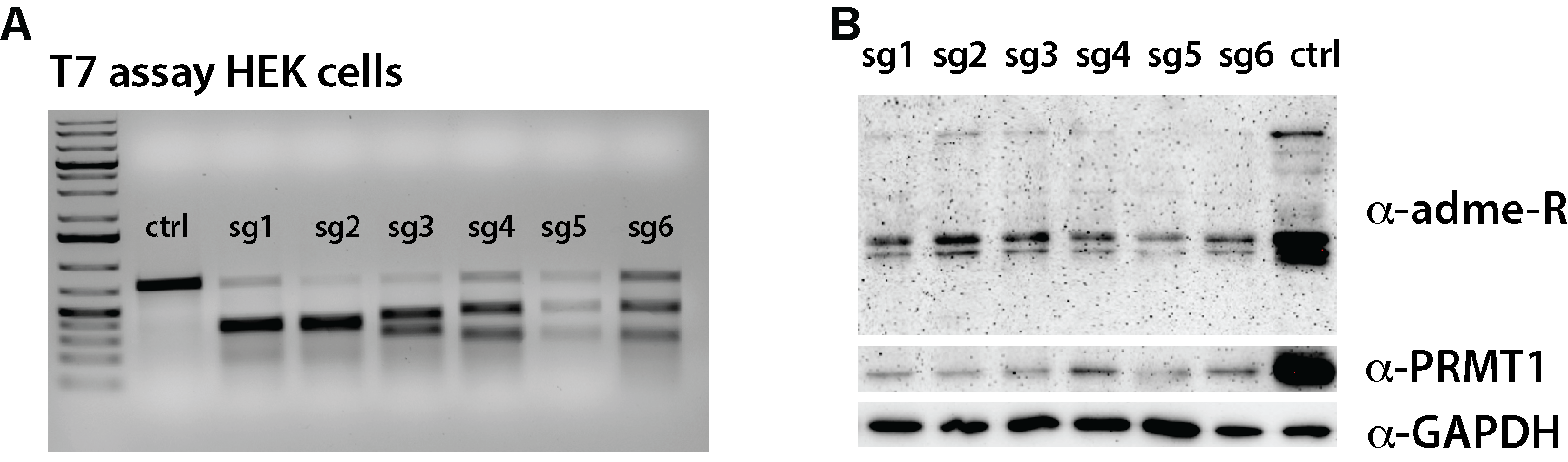
**

**Supplementary Figure 1.** Validation of sgRNAs targeting PRMT1. (A) T7E1 assay to test the cutting efficiencies of sgRNAs targeting PRMT1. Control (non-edited cells, ctrl) 6 sgRNAs (sg). (B) Western blot showing the efficiencies of PRMT1 deletion and total asymmetric di-methylation (α-adme-R) level upon knockout of PRMT1 by different sgRNAs. GAPDH was used as housekeeping gene.


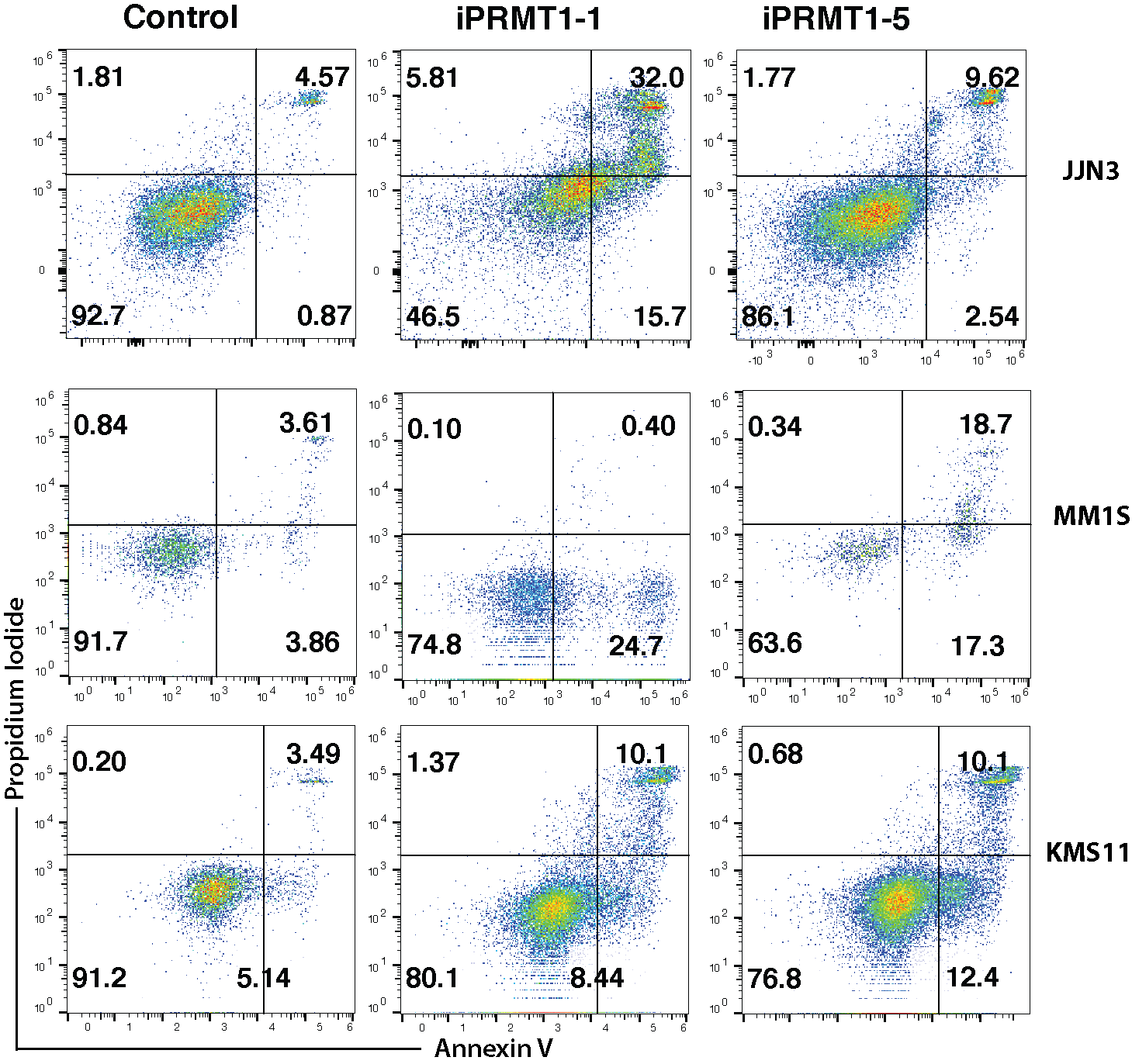


**Supplementary Figure 2.** Apoptotic assay. Control cells and PRMT1-KO cells by two different sgRNAs (4 days after supplementing Doxycycline) were stained with Annexin V and Propidium iodide (PI). Representative flow cytometry showing percentage of cell death.


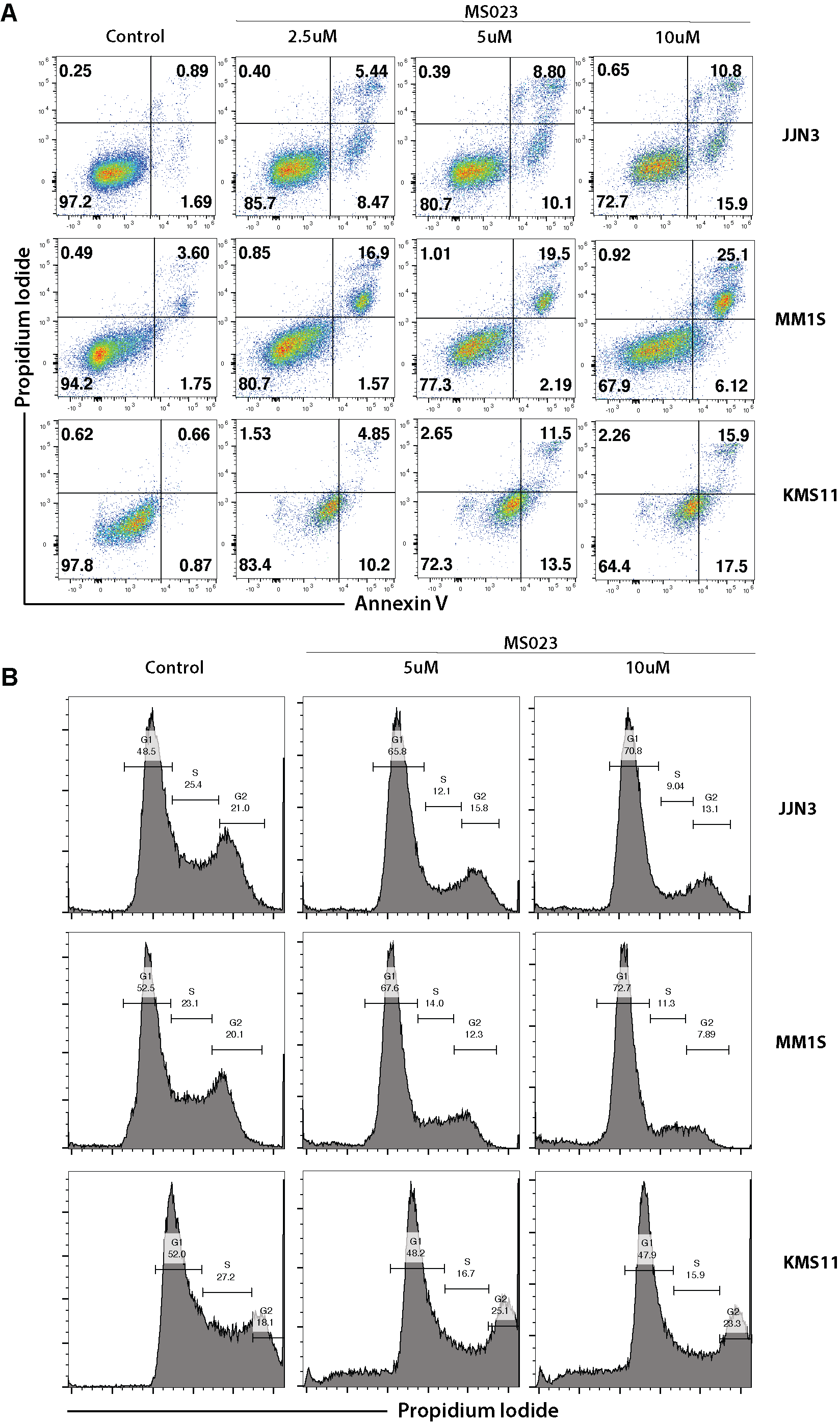


**Supplementary Figure 3.** (A) Three different MM cell lines were treated with MS023 with different concentrations for 3 days and stained with Annexin V and Propidium iodide (PI). Representative flow cytometry showing percentage of cell death. (B) Cell cycle profiles of MM cell lines treated with MS023 at different concentrations.


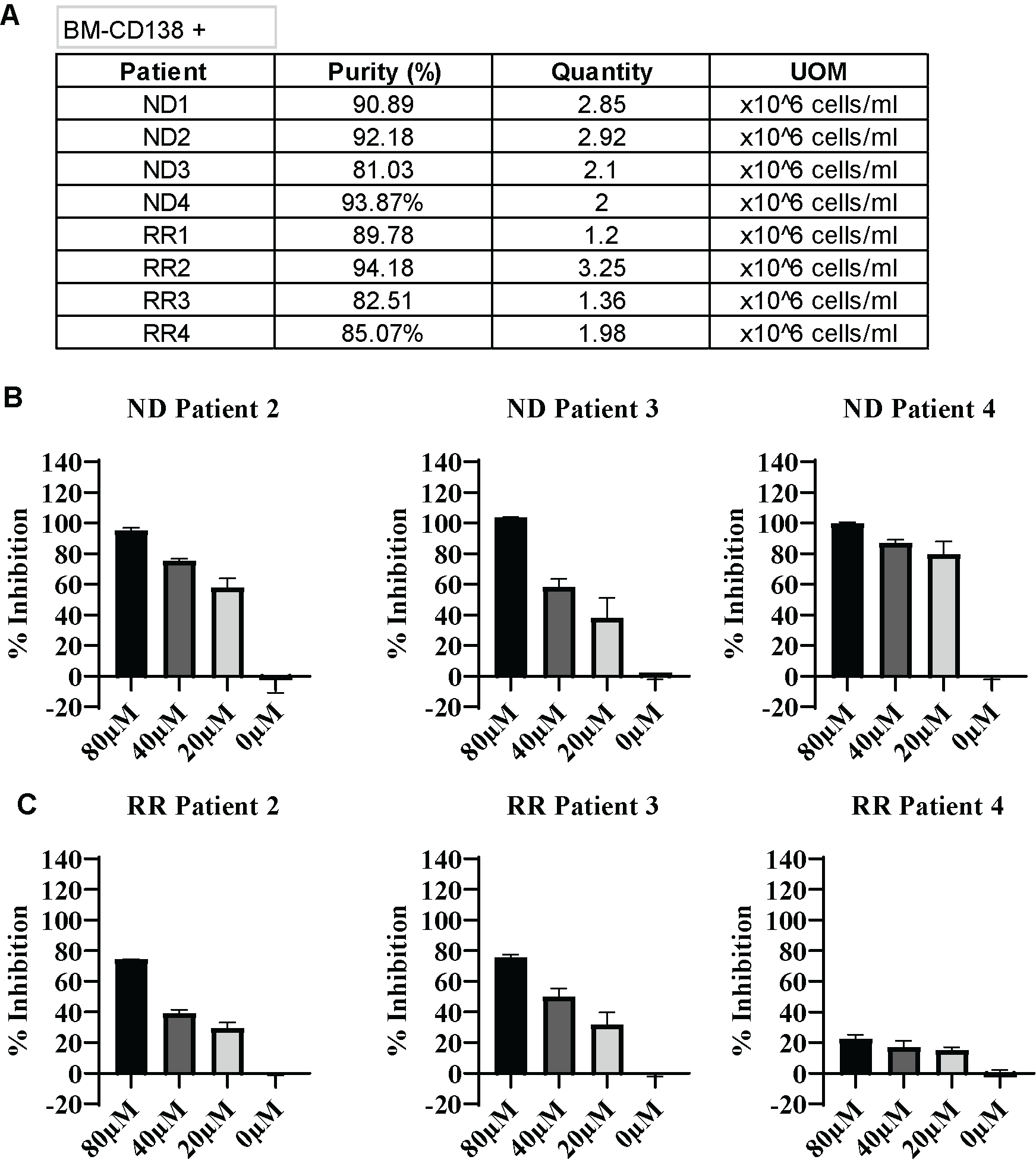


**Supplementary Figure 4.** Viability assay for primary MM cells from patients. (A) Table showing the purity of CD138+ cells from primary newly diagnosed (ND) and relapsed/refractory (RR) patients. Glo-Titer viability assay for ND patients (B) and RR patients (C) upon treated with MS023 at different indicated concentrations for 2 days. Data shows as mean value of technical triplicated.


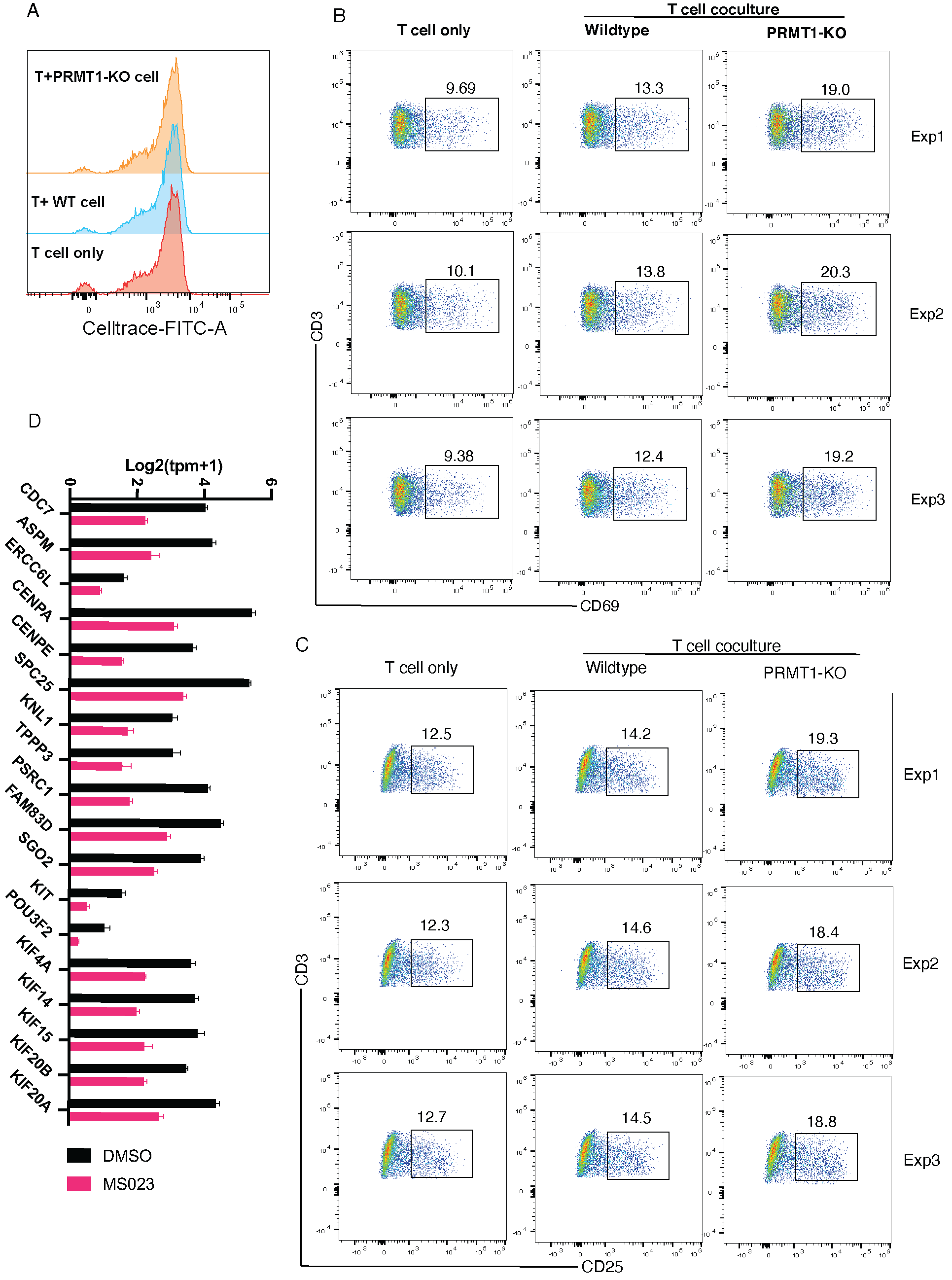


**Supplementary Figure 5.** T cell coculture with MM cell. JJN3 and JJN3-PRMT1-KO cells were irradiated and cocultured with activated human T cells (prestained with cell-trace dye). 48 hours later, cells were analyzed by flow cytometry to assess the proliferation (A) and the activation status of human T cells with CD69 (B) and CD25 (C). Cells were pre-gated with CD3+ cells. FACS plot shows the data of 3 independent experiments. (D) Expression of genes regulating cell division process extracted from RNA sequencing data.

**Supplementary Figure 6.** Non-contact T cell coculture with MM cell. JJN3 or JJN3-PRMT1-KO cells were seeded on the upper chamber of the transwell, while the activated human T cells were cultured in the bottom chamber. 48 hours later, cells were analyzed by flow cytometry to assess the the activation status of human T cells with CD69 (left) and CD25 (right). Cells were pre-gated with CD3+ cells. FACS plot shows the data of 3 independent experiments.

**Supplementary Figure 7.** Xenograft model for KMS11 cells. (A) tumor volumes and survival curves of mice transplanted with control KMS11 and PRMT1-KO KMS11 cells (n=5).
